# TiO_2_ NPs Assembled into a Carbon Nanofiber Composite Electrode by a One-Step Electrospinning Process for Supercapacitor Applications

**DOI:** 10.3390/polym11050899

**Published:** 2019-05-17

**Authors:** Bishweshwar Pant, Mira Park, Soo-Jin Park

**Affiliations:** 1 Department of Chemistry, Inha University, 100 Inharo, Incheon 22212, Korea; bisup@jbnu.ac.kr; 2 Department of Bioenvironmental Chemistry, College of Agriculture & Life Science, Chonbuk National University, Jeonju 561-756, Korea

**Keywords:** TiO_2_ nanofiber, TiO_2_-carbon, composite, electrospinning, supercapacitor

## Abstract

In this study, we have synthesized titanium dioxide nanoparticles (TiO_2_ NPs) into carbon nanofiber (NFs) composites by a simple electrospinning method followed by subsequent thermal treatment. The resulting composite was characterized by state-of-the-art techniques and exploited as the electrode material for supercapacitor applications. The electrochemical behavior of the as-synthesized TiO_2_ NPs assembled into carbon nanofibers (TiO_2_-carbon NFs) was investigated and compared with pristine TiO_2_ NFs. The cyclic voltammetry and charge–discharge analysis of the composite revealed an enhancement in the performance of the composite compared to the bare TiO_2_ NFs. The as-obtained TiO_2_-carbon NF composite exhibited a specific capacitance of 106.57 F/g at a current density of 1 A/g and capacitance retention of about 84% after 2000 cycles. The results obtained from this study demonstrate that the prepared nanocomposite could be used as electrode material in a supercapacitor. Furthermore, this work provides an easy scale-up strategy to prepare highly efficient TiO_2_-carbon composite nanofibers.

## 1. Introduction

The ever-increasing requirements for clean and sustainable energy have drawn intensive attention to the development of high-performance energy storage systems. Among the various electrochemical energy storage systems, the supercapacitors (SCs) are presumed as one of the most promising candidates for energy storage devices due to their superior performance in terms of power density and specific energy density, higher charge–discharge rate, and long cyclic life as compared to batteries and conventional capacitors [1,2]. Since the performance of the electrode materials governs the performance of the supercapacitor, the recent trend in research on supercapacitors has focused on the fabrication of highly efficient electrode materials with high energy density, which can maintain a high power density and cycling stability [1,3]. The electrodes of most of the supercapacitors are made up of carbon. However, the active electrode surface area and pore size distribution restrict the maximum capacitance [2]. One of the strategies to enhance the performance of electrode materials is to integrate metal oxides into the different carbon-based nanostructures in order to achieve the combined effect of the pseudocapacitance materials with a double-layer capacitor [1,4].

In the past few years, various transition metal oxides such as MnO_2_, ZnO, NiO, Fe_2_O_3_, Co_3_O_4_, MgO, and TiO_2_ (titanium dioxide) have been used along with carbon as a potential electrode material for supercapacitors [1,5]. Recently, TiO_2_-based nanostructures have been applied in electronic and optoelectronic devices due to their low cost, nontoxicity, eco-friendly nature, and abundant availability [6,7,8]. Due to its good electrochemical activity and high specific energy density, TiO_2_ can be considered as a promising candidate for a supercapacitor electrode [9,10]. Despite these advantages, the poor conductivity of TiO_2_ is the main obstacle that restricts its electrochemical performance [11]. Therefore, it is necessary to combine TiO_2_ with other materials that have good conductivity to form composites. The preparation of a TiO_2_-carbon composite can be an effective strategy to overcome the limitations of TiO_2_ to be used in a supercapacitor. In this regard, several TiO_2_-carbon composite nanostructures based mainly on carbon nanotubes (CNTs) and graphene have been prepared [7,12,13,14,15,16,17]. The composite showed better electrochemical performances; however, the synthesis procedure is complicated, and multiple steps are required. Furthermore, the use of toxic chemicals during the synthesis process also limits their widespread application. For example, the functionalization of CNT is generally carried out by the acid oxidation method using nitric acid (HNO_3_) and sulfuric acid (H_2_SO_4_) [18,19]. Similarly, the reduction of graphene oxide (GO) to reduced graphene oxide (RGO) is accomplished using chemical reducing agents, which may be toxic [20]. Several methods such as microwave, hydrothermal treatment, dip coating, chemical wet-impregnation, and electrospinning have been recognized as strategies for producing a TiO_2_-carbon composite [21,22,23,24]. Electrospinning has been considered as an effective method for producing good morphology of organic/inorganic nanofibers. However, it is important to find a cheap and eco-friendly procedure in order to synthesize the TiO_2_-carbon-based nanostructures for a supercapacitor electrode.

Recently, carbon nanofibers via an electrospinning process have gained extensive research interest due to their excellent conductivity, thermal and chemical stability, and easy fabrication process [25,26]. Mostly, carbon nanofibers are prepared from polyacrylonitrile (PAN) polymer. Besides PAN, various precursors, such as polyimide [27], cellulose [28,29,30], polyvinylidene fluoride (PVDF) [31], polyvinyl pyrrolidone (PVP) [32], and polyvinyl acetate (PVA) [33], have also been utilized to prepare carbon fibers via electrospinning followed by a suitable thermal treatment. The carbon fibers are highly applied as the electrode material in a supercapacitor due to their high operating voltage, attractive life span, and high capacity retention [34,35]. However, the specific capacitance and energy density need to be further enhanced in order to meet the growing energy demand. The introduction of metal oxides in the carbon nanofibers is beneficial for boosting the electrochemical properties of the resulting composite by the combined effect of the faradaic capacitance of the metal oxides and the double layer capacitance of the carbon nanofibers [1].

The composite of carbon nanofibers with TiO_2_ can be prepared easily by adding the modification additives in the precursor solution or TiO_2_ nanoparticles (NPs) formed earlier in the electrospinning process [1,32]. For example, Wang and coworkers [36] have synthesized TiO_2_ NP-decorated carbon nanofibers (NFs) from a P25/PAN solution by an electrospinning technique followed first by a heat treatment in air and then in a nitrogen environment. Similarly, Tang et al. [24] synthesized a TiO_2_-carbon NFs composite via an electrospinning technique by using a titanium oxycompound dispersed PAN/PVP solution. The as-fabricated nanocomposite showed good electrochemical performance [24]. The conversion of carbon fiber from PAN requires stabilization and carbonization processes under oxygen and inert atmosphere, respectively [24,25,37]. In this report, we have synthesized TiO_2_ NPs assembled into carbon nanofibers by an electrospinning process followed by a thermal treatment directly under an inert atmosphere and then investigated the performance of the as-synthesized nanocomposite in a supercapacitor. Importantly, this procedure simply requires the use of a polymer solution containing a TiO_2_ precursor. Upon thermal treatment, the prepared nanofibers simultaneously produce TiO_2_ NPs and carbon resulting in the formation of a TiO_2_ NP-embedded carbon nanofiber composite. We believe that the synthesis procedure is especially attractive since it offers a promising method for the assembly of TiO_2_ in carbon fibers and significantly boosts the electrochemical performance of the composite.

## 2. Experimental

### 2.1. Materials

Polyvinylpyrrolidone (PVP), acetic acid, and titanium tetraisopropoxide (TTIP, 97%) were purchased from Sigma-Aldrich (Seoul, Korea). Ethanol was purchased from Samchun Pure Chemicals, Co. Ltd., Seoul, Korea. All the chemicals were analytic grade and were used as received.

### 2.2. Synthesis of TiO_2_ NFs and TiO_2_-carbon NFs

**Figure 1:**
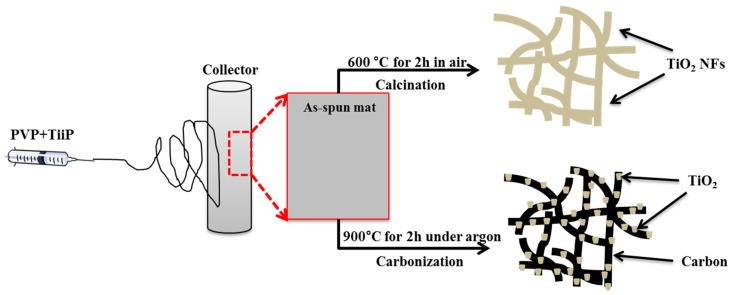
Synthesis protocol of pristine titanium dioxide nanofibers (TiO_2_ NFs) and TiO_2_-carbon NFs.

The synthetic protocol is given in Figure 1. TiO_2_ nanofibers were synthesized in the laboratory following our previous report [38]. Briefly, in the beginning, 1.5 g of TTIP was taken with 3 g of acetic acid in a vial and stirred for 10 min. Next, 0.5 g of PVP and 4 g of ethanol were added to the above mixture and stirred for 3 h. The solution was electrospun at 20 kV with a 15 cm distance from the tip to the collector. Two nanofiber mats were prepared under identical conditions and vacuum dried at 60 °C for 12 h. The nanofiber mats were subjected to thermal treatment under different conditions (air and argon). After calcination in air at 600 °C for 2 h, the TiO_2_ nanofibers were obtained. For the synthesis of the TiO_2_-carbon nanofiber composite, another mat was subjected to carbonization under the argon atmosphere at 900 °C for 2 h.

### 2.3. Characterization

The morphology of the prepared samples was checked with field-emission scanning electron microscopy (FE-SEM, Hitachi S-7400, Tokyo, Japan) and transmission electron microscopy (TEM, JEOL Ltd., Tokyo, Japan). The lattice structures were studied by X-ray diffractometer with Cu Kα radiation (λ = 0.15496 nm, Rigaku Co., Tokyo, Japan), scanned from 10 to 80°. The samples were characterized with Raman spectra by collecting on a Fourier transform infrared-Raman spectrometer (RFS-100S, Bruker, Hamburg, Germany). The Fourier transform infrared (FTIR) spectra were recorded using an ABB Bomen MB100 Spectrometer (Bomen, QC, Canada). The thermal behavior was studied by thermogravimetric analysis (TGA, Perkin-Elmer, Akron, OH, USA). For the TGA test, the samples were kept in a platinum pan located inside the furnace and heated from 25 to 800 °C under air flow at a heating rate of 10 °C/min.

### 2.4. Electrochemical Studies

**Figure 2:**
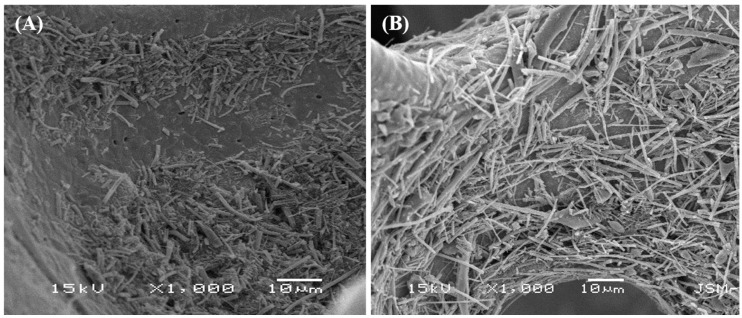
Scanning electron microscopy (SEM) image of the as-prepared slurry of titanium dioxide nanofibers (TiO_2_ NFs) (**A**) and TiO_2_-carbon NFs (**B**) with carbon black and polyvinylidene fluoride (PVDF) in *N*-methyl-2-pyrrolidone (NMP).

The working electrodes were prepared by coating the nickel foam with a homogenous slurry of as-prepared samples, carbon black, and polyvinylidene fluoride (PVDF) at a weight ratio of 8:1:1 in *N*-methyl-2-pyrrolidone (NMP). The substrate was then dried at 60 °C for 12 h. The morphology of the as-prepared slurry is given in Figure 2, which shows that the nanofibers were well distributed in the slurry. The electrochemical performance was investigated with a conventional three-electrode electrochemical analyzer at room temperature in a 2 M KOH solution as an electrolyte. Nickel foam, Ag/AgCl, and Pt wire were used as the working reference and counter electrodes, respectively. Cyclic voltammetry (CV), galvanostatic charge–discharge (GCD), electrochemical impedance spectroscopy (EIS), and the stability by charge–discharge cycles were performed using Iviumstat.

## 3. Results and Discussion

**Figure 3:**
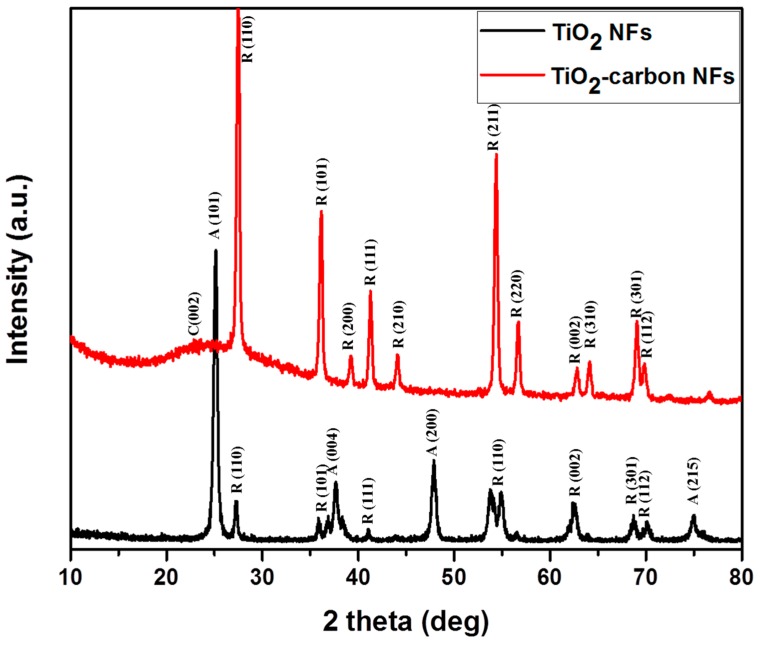
XRD spectra of as-synthesized TiO_2_-carbon NFs as compared to pristine TiO_2_ NFs.

Figure 3 shows the XRD patterns of the nanofibers synthesized under different calcination conditions. The nanofibers were found to be crystalline in both cases. As in the figure, the TiO_2_ nanofibers obtained by calcination at 600 °C in an air atmosphere possessed both anatase and rutile phases [32]. On the other hand, the TiO_2_-carbon composite fibers obtained by carbonization at 900 °C under an argon atmosphere consisted mainly of the rutile phase [32]. It is noteworthy to mention that the higher calcination temperature motivated the transformation of the crystal phase from anatase to rutile [39]. Furthermore, a broad peak located between the 2 theta values of 22° and 26° was observed, which is attributed to the (002) plane of the amorphous carbon [1]. Thus, the XRD suggests a successful conversion of PVP to carbon under the inert atmosphere. The crystal size of the as-synthesized TiO_2_ and TiO_2_-carbon composite fibers were calculated by employing the Debye–Scherrer equation with respect to their highly intensified peaks as below:

(1) D=kλ(βcosθ),

where “D” is the size of the crystal, “k” is a Scherer’s constant, “λ” is the wavelength of the X-ray, “β” is the full width at half maximum (FWHM) of the main peak, and “θ” is the diffraction angle. From the calculation, the obtained crystal sizes for TiO_2_ NFs and TiO_2_-carbon NFs were 25.11 and 27.48 nm, respectively.

**Figure 4:**
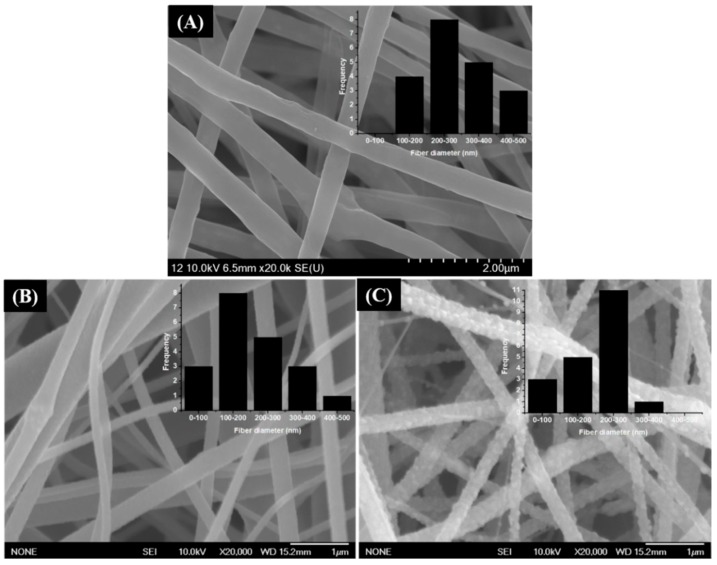
Field-emission scanning electron microscopy (FE-SEM) images of an as-spun nanofiber membrane (**A**), pristine TiO_2_ NFs (**B**), and TiO_2_-carbon NFs (**C**). The insets show their corresponding diameter distributions.

**Figure 5:**
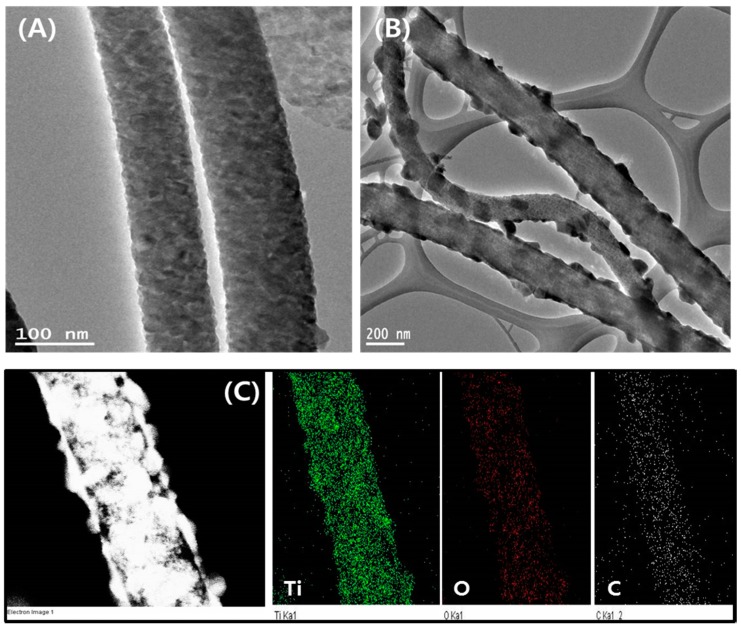
Transmission electron microscopy (TEM) images of pristine TiO_2_ NFs (**A**) and TiO_2_-CNFs (**B**). (**C**) shows the elemental mapping of the TiO_2_-carbon NF samples.

Figure 4A–C depict the morphologies of the as-spun fiber membrane, TiO_2_ NFs, and TiO_2_-carbon NFs, respectively. The as-spun nanofibers showed continuous and bead-free morphology with an average fiber diameter of 290 ± 130 nm (Figure 4A). Well-preserved nanofibrous morphology was obtained after the thermal treatment in both cases. TiO_2_ nanofibers showed smooth and continuous morphology. It is noticeable that during the calcination process, the PVP was selectively removed and continuous fibers of TiO_2_ were obtained (Figure 4B). The calcination under the inert atmosphere resulted in the formation of TiO_2_-incorporated carbon nanofiber structure. During the calcination under the inert atmosphere, PVP was graphitized and titanium tetraisopropoxide was decomposed to a stable TiO_2_ form, leading to the production of TiO_2_-carbon composite fibers (Figure 4C). TiO_2_ NFs showed an average fiber diameter of 250 ± 170 nm, while the TiO_2_-carbon NFs showed an average diameter of 160 ± 115 nm. The difference in diameter distribution is attributed to the atmospheric condition during the thermal treatment. The internal structure of the as-prepared samples was studied by transmission electron microscopy (TEM). As in the figure, the pure TiO_2_ NFs revealed a smooth and uniform fibrous morphology (Figure 5A), whereas the composite nanofibers were comprised of carbon nanofibers embedded with TiO_2_ nanocrystals (Figure 5B). The existence of carbon nanofibers and TiO_2_ in the composite was also confirmed by the mapping images (Figure 5C).

**Figure 6:**
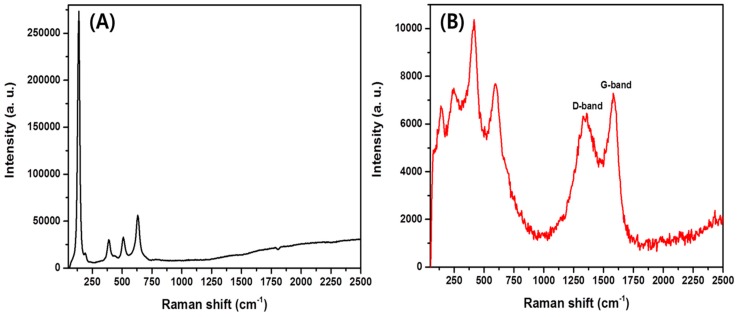
Raman spectra of TiO_2_ NFs (**A**) and TiO_2_-carbon NFs (**B**).

Figure 6 shows the Raman spectra of pristine TiO_2_ NFs and TiO_2_-CNFs. As in Figure 6A, the pristine TiO_2_ NFs are rich in the anatase phase showing major peaks at 142, 388, 516, and 638 cm^−1^ [36,40,41]. After calcination under argon atmosphere at 900 °C (Figure 6B), the major anatase peak at 142 was highly suppressed and other peaks disappeared. Instead, three new peaks appeared at about 245, 420, and 600 cm^−1^, which were ascribed to the rutile phase of TiO_2_ [36,40]. These changes indicate a transformation of the anatase to rutile phase upon a higher degree of thermal treatment. Besides the TiO_2_ peaks, two broad peaks corresponding to the D-band and G-band of carbon were observed around 1350 and 1590 cm^−1^, respectively, which further proves that the PVP had been converted to the carbon structure [25]. The results obtained from Raman are consistent with the XRD data.

**Figure 7:**
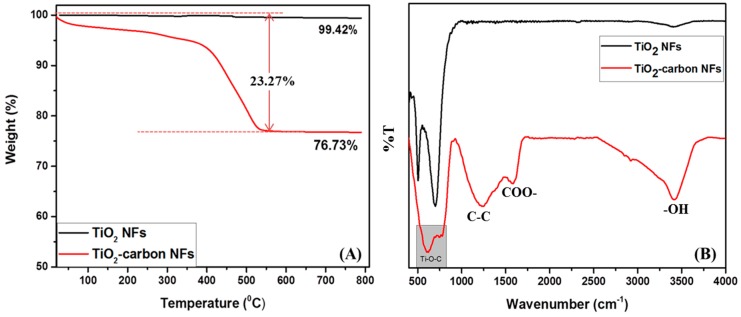
Thermogravimetric analysis (TGA) (**A**) and Fourier transform infrared spectroscopy (FTIR) spectra (**B**) of the TiO_2_-carbon NFs as compared to the TiO_2_ NFs.

Figure 7A represents the thermogravimetric analysis (TGA) profiles of the samples. The pristine TiO_2_ NFs show high thermal stability (~99.42% at 800 °C). In the case of the composite sample, a gradual decrease in the weight was observed until 550 °C and after that temperature, there was no weight loss. Since TiO_2_ did not decompose at the given temperature, the weight loss (23.27%) could be attributed to the loss of the carbon content in the composite sample. From the obtained data, the carbon and TiO_2_ contents in the composite sample were estimated to be ~23.27% and ~76.73%, respectively. The interaction between the TiO_2_ and carbon was studied by Fourier transform infrared spectroscopy (FTIR) and the results are given in Figure 7B. The pristine TiO_2_ NFs showed absorption peaks around 500–700 and 3400 cm^−1^ for Ti–O vibration and O–H bending of water molecules absorbed, respectively [42]. In the case of TiO_2_-carbon composite nanofibers, the peaks at about 1200 and 1550 cm^−1^ are attributed to the C–C stretching vibration and asymmetric and symmetric stretching band of COO–, respectively [1]. The presence of Ti–O–C bonds around 500–750 cm^−1^ represents the interaction of TiO_2_ with the carbon, suggesting the successful formation of the composite [6,23].

**Figure 8:**
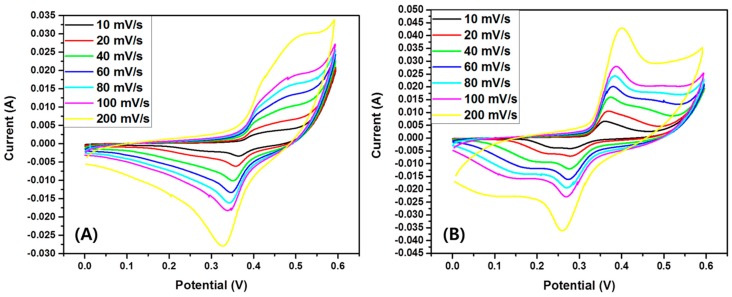
Cyclic voltammetry (CV) curves of pristine TiO_2_ NFs (**A**) and TiO_2_-carbon NFs (**B**) at different scan rates.

The electrochemical performance of the as-synthesized TiO_2_-carbon NFs compared with the pristine TiO_2_ NFs was evaluated by CV, GCD, and EIS tests in a three-electrode system electrochemical cell by using 2 M KOH as an electrolyte. Figure 8A,B show the CV curves of pristine TiO_2_ and TiO_2_-carbon NF samples at different scan rates from 10 to 200 mV/s with a potential window from 0 to 0.6 V, respectively. As in the figure, the CV curve of TiO_2_-carbon NFs shows a larger area compared to that of pristine TiO_2_ NFs, suggesting a better capacitive behavior. The quasi-rectangular shape of all curves clearly indicates pseudocapacitive characteristics. The slight difference in the redox peaks of the composite compared to the pristine TiO_2_ NFs may be due to the structural difference in the two samples. It is noteworthy to mention that the pristine TiO_2_ NFs were composed of both rutile and anatase phases, whereas the TiO_2_-carbon NFs were composed mainly of the rutile phase. In addition, the TiO_2_-carbon sample could have the combined effect of the faradaic capacitance of the TiO_2_ and the double-layer capacitance of the carbon.

**Figure 9:**
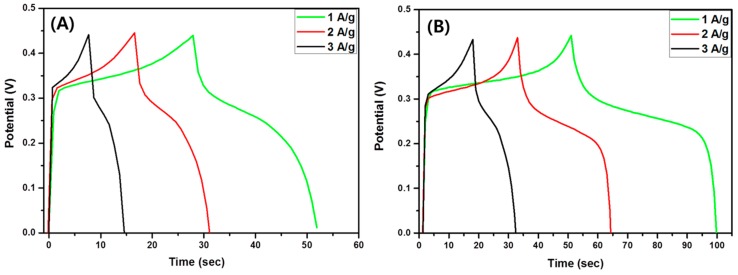
Galvanostatic charge–discharge (GCD) curves of pristine TiO_2_ NFs (**A**) and TiO_2_-carbon NFs (**B**) at different current densities.

**Figure 10:**
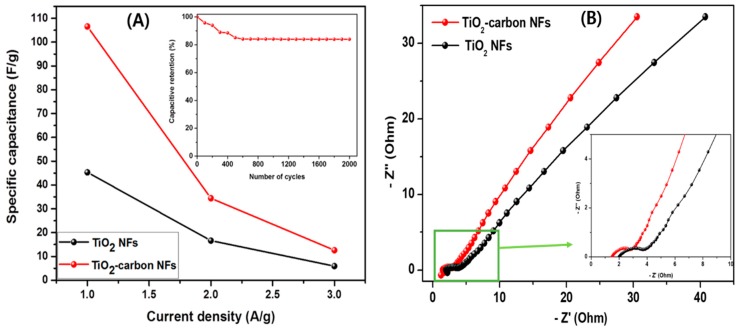
The specific capacitance (**A**) and electrochemical impedance spectroscopy (EIS) test (**B**) of different electrodes. Inset A and B represent the stability of the TiO_2_-carbon NF composite and semicircle area in the EIS spectra, respectively.

The specific capacitance of the composite material was quantified by GCD studies over the potential window between 0 and 0.45 V at various current densities (1, 2, and 3 A/g) as shown in Figure 9. It can be seen that when the current density was increased, the discharge time of the material decreased significantly. This could be due to the sluggish kinetics of the redox reaction to fast potential change [5]. Many factors such as specific surface area, pore size, and conductivity can affect the specific capacitance of the electrode materials [43]. The specific capacitance of the electrodes was calculated by the equation below:

(2) $$C=\frac{I\cdot \;\Delta t}{m\cdot \;\Delta V}\;,$$

where “*C*” is the specific capacitance (F/g), “*I*” is the charge–discharge current (A), “*t*” is the discharge time (s), “*m*” is the mass of the active material (g), and “*V*” is the potential window (V). The potential window in the charge–discharge curves match with the cyclic voltammetry curves and suggest that the specific capacitance of the materials is due mostly to the faradaic reaction [1]. The specific capacitances of TiO_2_ NFs were 45.31, 16.62, and 5.92 F/g at the current densities of 1, 2, and 3 A/g, respectively (Figure 10A). As expected, the specific capacitance values were found to be increased in the case of TiO_2_-carbon NFs. The specific capacitance of the TiO_2_-carbon NF composite was calculated to be 106.57, 34.44, and 12.59 F/g at the current densities of 1, 2, and 3 A/g, respectively (Figure 10A). It is obvious that the capacitance of the electrode decreased with increasing current densities, which was due to the kinetic resistance and insufficient charge transfer across the electrode–electrolyte interface at higher current densities [5,43]. A drop in the discharge curve in the case of pristine TiO_2_ NFs was noticed, which could be due to the equivalent series resistance (ESR). As compared to the pristine TiO_2_ NFs, the composite showed better performance. The electron-transfer kinetics of the electrode materials were studied by EIS analysis (Figure 10B). The smaller semicircular arc in the high-frequency range in the Nyquist plots of the TiO_2_-carbon NF composite as compared to the pristine TiO_2_ NFs (inset in Figure 10B) shows a better charge transfer property than that of the pristine TiO_2_ NFs [1,44]. The inset in Figure 10A displays cyclic stability of the TiO_2_-carbon NF composite. As in the figure, it revealed about 84% capacitance retention after 2000 cycles (Figure 10A; inset). A comparison of the electrochemical performance of the as-synthesized TiO_2_-carbon composite nanofibers with some previously investigated electrodes is given in Table 1, which indicates a satisfactory performance of the as-prepared TiO_2_-carbon NF composite for supercapacitor applications.

## 4. Conclusions

TiO_2_ NPs embedded into carbon nanofibers were successfully prepared by an electrospinning technique and post thermal treatment under argon atmosphere. Upon heating at 900 °C, the anatase to rutile phase transformation was achieved. The loading of TiO_2_ into the amorphous carbon showed an extraordinary enhancement in terms of electrochemical properties. The cyclic voltammetry, galvanostatic charge–discharge, and EIS test results exhibited a combined synergistic effect of TiO_2_ and carbon fibers. Furthermore, the higher conductivity of the rutile phase and lower charge limitation also favored the electrochemical property enhancement. Overall, the results obtained from the electrochemical study and the easy synthesis protocol show potential application in several applications, including supercapacitors.

## Figures and Tables

**Table 1:** Comparison of the electrochemical performance of as-synthesized TiO_2_/carbon composite nanofibers for supercapacitor applications with some investigated electrodes.

S. N.	Electrode Material	Fabrication Method	Electrolyte Used	Specific Capacitance	Stability	Ref.
1	TiO_2_-activated carbon	Microwave	0.1 N Na_2_SO_4_	92 F/g (at 5 mV/s)	~89% (5000 cycles)	[22]
2	rGO/TiO_2_/rGO	Hydrothermal	1 M Na_2_SO_4_	64.3 F/g (at 0.1 mA/cm^2^)	85% (4000 cycles)	[21]
3	BC-G-TiO_2_	Dip coating and high temperature treatment	1 M H_2_SO_4_	250.8 F/g (at 2 A/g)	84.4% (100 cycles)	[45]
4	TiO_2_-CNT	Chemical wet-impregnation	1 M H_2_SO_4_	110 F/g (at 0.05mA/cm^2^)	-	[46]
5	Fe-TiO_2_/C nanofibers	Electrospinning	1 M KOH	137 F/g (at 5 mV/s)	-	[47]
6	MPTNF/rGO	Electrospinning	1 M H_2_SO_4_	210.5 F/g (at 1 A/g)	97% (1000 cycles)	[13]
7	TiO_2_@CNFs	Electrospinning	6 M KOH	151.5 F/g (at 1A/g)	97.8% (4000 cycles)	[24]
8	TiO_2_-carbon nanofibers	Electrospinning	2 M KOH	106.57 F/g (at 1A/g)	84% (2000 cycles)	This study
